# Neuropsychological effects of cannabis use by continent and age: a systematic review

**DOI:** 10.3389/fpsyg.2026.1728743

**Published:** 2026-05-28

**Authors:** Lidia Venero-Hidalgo, Francisca Carvajal, Fernando Rodríguez de Fonseca, Víctor José Villanueva-Blasco

**Affiliations:** 1 Department of Psychology, Faculty of Psychology, University of Almería, Almería, Spain; 2 Health Research Center CEINSA, University of Almería, Almería, Spain; 3 Clinical Neurology Unit, Institute of Biomedical Research of Malaga (IBIMA), Regional University Hospital of Malaga, Málaga, Spain; 4 Faculty of Health Sciences, Valencian International University, Valencia, Spain

**Keywords:** adolescence, age, attention, cannabis, cognition, geographical difference, memory, young adult

## Abstract

**Introduction:**

Cannabis use during adolescence and young adulthood has been associated with neurocognitive alterations. However, limited attention has been paid to whether these findings vary according to developmental stage and regional context. This systematic review examined the neurocognitive effects of cannabis use in adolescents and young adults, with particular attention to age-related patterns and geographic variation.

**Methods:**

Following PRISMA guidelines, a systematic search was conducted in PubMed/MEDLINE, Scopus, Web of Science, and OpenGrey for studies published between 1 January 2019 and 30 June 2024. Eligible studies assessed cognitive performance in adolescent or young adult cannabis users. Methodological quality was appraised using the Mixed Methods Appraisal Tool, and certainty in the body of evidence was evaluated using the Grading of Recommendations Assessment, Development and Evaluation approach.

**Results:**

Twenty-two studies met the inclusion criteria. Across the included studies, memory-related impairments were the most consistent findings, particularly in episodic and working memory. Evidence regarding executive functions was more heterogeneous and appeared to vary according to age group, exposure profile, study design, and cognitive task. Studies from the Americas, many of them longitudinal, more often reported negative associations between cannabis use and cognitive performance. European findings were more mixed, possibly reflecting differences in product characteristics, co-use patterns, and measurement approaches. Evidence from Asia was limited.

**Discussion:**

Overall, the findings suggest that cannabis-related neurocognitive differences are more consistently detected in adolescent samples than in young-adult samples, although this pattern is likely shaped by developmental, methodological, and contextual factors. Future research should improve comparability in exposure definitions, control of confounding variables, and selection of cognitive measures.

## Introduction

Cannabis is among the most widely consumed psychoactive substances globally, with approximately 228 million users in 2022 (Duperrouzel et al., 2020; United Nations Office on Drugs and Crime, 2024). Its use has been proposed to be linked to numerous health risks, including mental health problems, immune suppression, cancer, and cardiovascular and respiratory diseases (Chandy et al., 2024; Liu et al., 2020). Some studies suggest that chronic cannabis use may also cause structural and functional brain changes, especially in the hippocampus, amygdala, and prefrontal cortex (Cabeen et al., 2020; Ogunbiyi et al., 2020; Rivera-Olmos and Parra-Bernal, 2016), contributing to cognitive deficits in memory, attention, inhibition, and executive functions (Venero-Hidalgo et al., 2022), and ultimately accounting for associated mental health problems.

The severity of these effects depends largely on consumption patterns, particularly age of onset, which is a strong predictor of cognitive impairment and mental health issues (Fonseca-Pedrero et al., 2020; Gorey et al., 2019). Initiating cannabis use during adolescence, a period marked by active brain development (Duperrouzel et al., 2020), significantly increases the risk of addiction (Fonseca-Pedrero et al., 2020), and may interfere with neurodevelopmental processes regulated by the endocannabinoid system (Lu and Mackie, 2021). Cannabis use during adolescence has been associated with disrupted synaptic pruning (Bossong and Niesink, 2010) and reduced gray matter volume in CB1-rich regions such as prefrontal cortex and hippocampus (Rojas Bernal et al., 2024; Solowij et al., 2011), disrupting networks critical for cognitive and emotional regulation (Mena et al., 2013).

Although cannabis use is prevalent worldwide, patterns of consumption vary substantially across regions and may shape the neurocognitive effects observed in different study settings (Rafei et al., 2023). In North America, cannabis is more often used without tobacco (Hindocha et al., 2016; Substance Abuse and Mental Health Services Administration, 2025), whereas in Europe it is frequently mixed with tobacco (European Union Drugs Agency, 2024; European Union Drugs Agency, 2025; Gravely et al., 2020; Hindocha et al., 2016; Villanueva-Blasco et al., 2024a). This distinction is important because tobacco use itself has been associated with impairments in cognitive domains such as attention, inhibitory control, and decision-making (Chamberlain et al., 2012; Heishman et al., 2010), and dual cannabis–tobacco use has been linked to greater health risks and a higher likelihood of dependence than cannabis use alone (Agrawal et al., 2012; Gravely et al., 2020). However, relatively few studies have distinguished the neurocognitive effects of cannabis-only use from those associated with co-use involving tobacco.

At the same time, regional differences in neurocognitive findings are likely to reflect more than cannabis–tobacco co-use alone. Cannabis markets also differ in THC concentration, THC/CBD balance, product type (e.g., herbal cannabis, resin, concentrates, vaping products, edibles), route of administration, age of onset, repeated-use patterns, and regulatory context, all of which may influence dose, frequency of exposure, and cumulative neurocognitive burden (European Union Drugs Agency, 2024; European Union Drugs Agency, 2025; National Academies of Sciences, Engineering, and Medicine et al., 2024; Rafei et al., 2023).

Because these contextual factors are rarely considered in a systematic way, and because previous reviews have not consistently incorporated regional patterns of use into their interpretations (Blest-Hopley et al., 2019; Duperrouzel et al., 2020; Gorey et al., 2019; Venero-Hidalgo et al., 2022), the geographical contextualization of findings is essential for interpreting apparent cross-national differences in cannabis-related neurocognitive outcomes.

Accordingly, this study examines the available evidence on the neurocognitive effects of cannabis use in adolescents and young adults, with particular attention to developmental stage and regional context. Specifically, it aims to: (1) synthesize evidence on cognitive and executive-function outcomes associated with cannabis use; (2) examine whether reported findings vary according to age group and geographic setting; and (3) assess the methodological quality of the included studies and the certainty of the overall evidence. The guiding questions were: What neurocognitive outcomes have been associated with cannabis use in adolescents and young adults? To what extent do reported findings vary by age and region? How robust is the available evidence, and what methodological factors may explain inconsistencies across studies?

## Methods

### Search strategy and information sources

This systematic review was conducted in June 2024 in accordance with the Preferred Reporting Items for Systematic Reviews and Meta-Analyses (PRISMA) guidelines (Page et al., 2021) and was registered in PROSPERO (CRD42024556357). The search covered studies published between 1 January 2019 and 30 June 2024.

The search strategy was developed using the Problem–Intervention–Outcomes (PIO) framework (Stone, 2002) and was applied to four sources: PubMed/MEDLINE, Web of Science, Scopus, and OpenGrey. Search terms combined cannabis-related keywords (e.g., *cannabis*, *marihuana*, *marijuana*, *THC*), cognitive and neuropsychological terms (e.g., *cogniti* *, *neuropsycholog* *, *attention*, *working memory*, *executive function*, *shifting*, *switching*, *decision making*), and age-related terms (e.g., *adolescent*, *young adult*). In PubMed/MEDLINE, the search combined free-text terms in the title/abstract fields with controlled vocabulary terms (MeSH), whereas the search strategies in Web of Science and Scopus were adapted to the syntax of each database. A simplified search string was used in OpenGrey. The full database-specific search strategies are presented in Appendix Table A1.

To reduce publication bias, one grey-literature source was also searched. OpenGrey was retained because, although it is no longer actively updated, its archived records remain accessible and may still contain relevant material. No eligible studies were ultimately identified through this source.

### Eligibility criteria

The screening process applied predefined inclusion and exclusion criteria. Eligible studies had to: (a) be peer-reviewed articles published in English or Spanish; (b) have been published between 1 January 2019 and 30 June 2024 to ensure that findings reflect the evolving regulatory context (National Academies of Sciences, Engineering, and Medicine et al., 2024) and increased potency of cannabis products (European Union Drugs Agency, 2024); (c) assess neuropsychological or cognitive performance outcomes in relation to cannabis use; and (d) focus on adolescents, young adults, or mixed samples spanning late adolescence and emerging adulthood.

For the purposes of synthesis, age-group membership was defined at the study-sample level rather than by reclassifying individual participants. Studies were coded as adolescent when the original sample was described by the authors as adolescent and the age range was primarily within adolescence (11–17 years in the studies included here); as young adult when the sample was primarily aged 18 years or older; and as mixed adolescent/young adult when the recruited range crossed the late-adolescent/emerging-adult boundary or when separate adolescent and adult subsamples were included within the same study (e.g., 14–22 or 16–29 years). Because the primary studies used heterogeneous age boundaries, the original authors’ developmental framing was preserved and this categorization was used only for narrative synthesis.

Studies were excluded if they involved non-human subjects, medical cannabis use, systematic reviews or meta-analyses, or if polysubstance exposure prevented the specific neurocognitive effects of cannabis from being interpreted. Studies were also excluded when major comorbid neuropsychiatric conditions made the population non-comparable to the review question. However, studies were not automatically excluded if variables such as alcohol use, nicotine/tobacco use, depression, or anxiety were measured and handled analytically, balanced between groups, or otherwise incorporated into the study design.

At full-text screening, “wrong population” referred to studies not focused on adolescents or young adults, studies involving medical cannabis populations, or studies with major clinical characteristics outside the target population. “Wrong outcomes” referred to studies that did not report cognitive or neuropsychological outcomes based on task performance or structured cognitive assessment, including studies focused exclusively on prevalence, attitudes, psychiatric symptoms, or non-cognitive outcomes.

### Data extraction

Study selection and data extraction were carried out independently by two researchers (LVH and FC). Each reviewer assessed full texts against the eligibility criteria, and disagreements were resolved by consultation with a third researcher (VJV-B).

**Figure 1:**
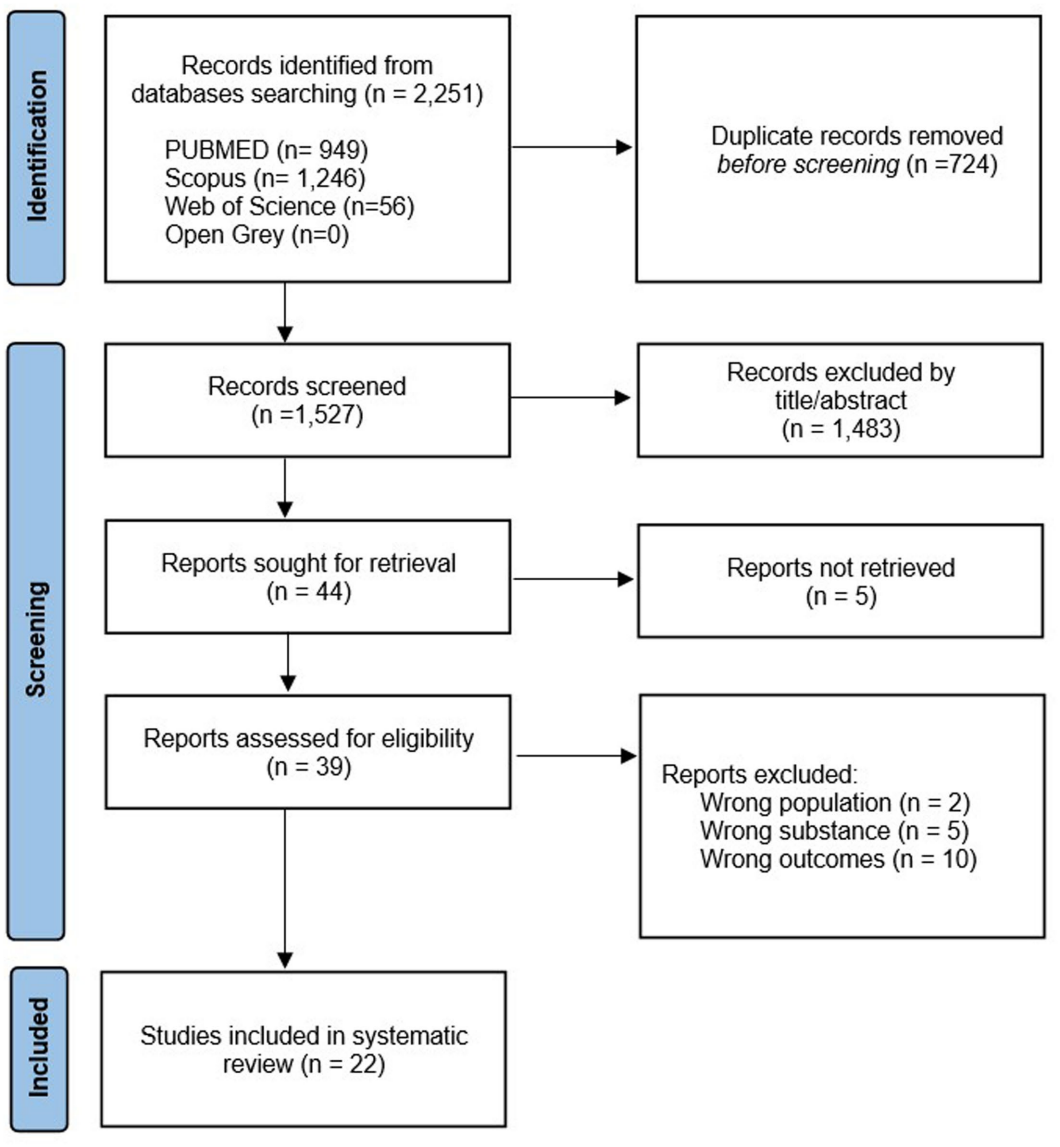
PRISMA flow diagram of the study selection process.

In addition to bibliographic information and study design, the following data were extracted from each study: sample size, age range, region, cannabis exposure profile (e.g., occasional/light, frequent, chronic, acute intoxication, abstinent users), comparator type, abstinence window when reported, handling of alcohol/nicotine/tobacco or other confounders, and the type of cognitive assessment used (brief screening measure, standardized neuropsychological battery, computerized performance-based task, or self-report proxy). The details of the selection process are summarized in Figure 1, and the characteristics of the included studies are presented in Table 1.

**Table 1:** Characteristics of the included studies in chronological order.

Authors (year)	Geographical area	Study design	Participants	Comparison of cannabis exposure profile	Cognitive assessment type	Test	Co-use/confounder handling	Results
Macedo et al. (2024)	Europe	Longitudinal study	2,341 adolescents and young adults (14-22 years)	Light users vs. controls	Standardized tests and questionnaires	ESPAD, AUDIT, FTND, SDQ, WISC IV, WAIS IV, SDT and a scale assessing family stresses	AUDIT and FTND measured; conduct problems and family stress considered	Baseline cognitive functioning (age 14) did not predict future cannabis use. Conduct problems at baseline predicted the cannabis use at age 19. No significant differences in cognitive functioning (verbal comprehension, visuospatial function and working memory) between light cannabis users and controls.
Wade et al. (2024a)	America	Cross-sectional study	129 current cannabis users and 96 controls (16-22 years)	Users vs. controls	Performance-based battery	Emotional and Word-Emotional Face Stroop, Dimensional Change Card Sort Test, Flanker inhibitory Control and List Sort Working Memory Test	Age of onset and sex subgroup analyses; co-use not clearly reported	Working memory functioning was modestly associated with past-6 month cannabis use. Small reduction in processing speed on an emotional task in cannabis users. No differences in inhibitory control and set-shifting were observed between groups. There were no significant relationships between age of onset and sex subgroups and cognitive outcomes.
Wade et al. (2024b)	America	Cross-sectional study	123 cannabis users and 123 non-using controls (13-14 years)	Users vs. non-users	Performance-based battery	Picture Sequence Memory Test, Picture Vocabulary Test, List Sorting Working Memory Test, Flanker Inhibitory Control Test, Pattern Comparison Process Speed Test, and Oral Reading Test	Exposure assessment strengthened by hair toxicology; other confounders not clearly reported	Lower scores on an episodic memory task in cannabis users. More cannabis use was linked to poor performances on verbal, inhibitory, working memory and episodic memory tasks.
Block et al. (2022)	America and Europe	Cross-sectional study	23 regular marijuana users and 25 controls (12-19 years)	Regular users vs. controls; regional subsamples	Standardized neuropsychological tests	BSRT and WCST	Regional subgroup comparison; co-use/confounders not clearly reported	Regular marijuana users showed lower overall long-term storage in BSRT. Marijuana was associated with memory effects only among American users. Lower total recall and more perseverative errors in BSRT were associated with marijuana use in the last year.
Goud et al. (2022)	Asia	Observational case–control study	50 active cannabis users and 50 controls (18-25 years)	Users vs. controls; baseline vs. 3-month abstinence	Brief cognitive screening	MoCA	Duration and quantity considered; co-use/confounders not clearly reported	Differences between the baseline MoCA score of cannabis users and the controls. Duration and quantity of cannabis use had a negative correlation with the MoCA score. Partially improvement in cognitive functioning in cannabis users after 3 months of abstinence.
Kroon et al. (2024)	Europe	Cross-sectional study	104 frequent cannabis users and 85 never to sporadic users (18-25 years)	Frequent users vs. never/sporadic users	Performance task and questionnaires	N-back task, CUDIT-R, AUDIT, FTND, WAIS-IV, BDI-II and STAI	Alcohol, nicotine, depression, and anxiety measured	Cannabis users showed a more pronounced decrease in performance (accuracy and time reaction) as the difficulty of an N-back WM task increased. Smaller reduction in activity in the precuneus and PCC in cannabis uses at higher WM load.
Lawn et al. (2022a)	Europe	Randomized controlled trial	48 adolescents and young adults (16-29 years)	Experimental exposure/age comparison	Experimental task battery	Subjective ‘Feel drug effect’, delayed prose recall task and PSI	Experimental design; co-use/confounders not clearly reported	Verbal episodic memory impaired in adolescents after cannabis exposure. No age differences in acute psychotomimetic effects, neither related to verbal memory nor to subjective effects caused by cannabis use.
Lawn et al. (2022b)	Europe	Cross-sectional study	274 participants: 76 cannabis users and 63 controls (16-17 years) and 71 adult cannabis users and 64 adult controls (26-29 years)	Users vs. controls within age strata	Performance-based tasks	The prose recall task, the spatial n-back task and stop-signal task	Age-stratified comparison; co-use/confounders not clearly reported	Cannabis users had worse verbal episodic memory. No differences on spatial working memory or response inhibition between groups.
Pacheco-Colón et al. (2022)	America	Longitudinal study	401 adolescents (14-17 years)	Within-cohort exposure gradients	Standardized battery and decision-making tasks	SCID-II, IGT, GDT and CT, Wechsler Memory Scale IV, CVLT-II, Drug Use History Questionnaire and Structured Clinical Interview for DSM-IV	Drug history and clinical interview used; several confounders considered	Frequency of cannabis use was associated with poorer episodic memory at baseline. Greater escalation of cannabis use predicted lesser gains in immediate episodic memory. Decision-making did not predict escalation or initiation of cannabis use.
Wiedmann et al. (2022)	Europe	Cross-sectional study	Nine chronic cannabis users and nine without (15-17 years)	Chronic users vs. non-users	Standardized neuropsychological tests	VLMT, TAP Go/NoGo, Stroop, and Stop-Signal	Extent of use examined; very small sample; co-use/confounders not clearly reported	No differences in attentional performance or inhibitory control was observed between the groups. Higher extent of chronic cannabis use was associated with lower scores in verbal learning.
Ajmera et al. (2021)	America	Prospective longitudinal study	18 frequent cannabis users and 29 infrequent cannabis user (13-19 years)	Frequent/moderately frequent vs. infrequent users	Standardized neuropsychological tests	RAVLT and IGT	Frequency-based comparison; co-use/confounders not clearly reported	Moderately frequent cannabis users showed pre-initiation decrements in new learning performance. Declines in total verbal learning and delayed recall over time in moderately frequent cannabis users. No significant differences between groups were detected in decision-making performance.
Frolli et al. (2021)	Europe	Cross-sectional study	100 chronic users of cannabis, 100 with occasional use of cannabis and 100 controls (15-16 years)	Chronic vs. occasional vs. controls	Standardized neuropsychological battery	WISC IV, Tower of London, Corsi Test from the BVN, and the MT Trials-Advanced 3	Exposure severity differentiated; co-use/confounders not clearly reported	Chronic cannabis use had a significant impact on verbal comprehension index, visual-perceptive reasoning, working memory functions, processing speed, planning and visuospatial memory skills. Occasional cannabis use impaired performance of working memory. Chronic users showed worse IQ, working memory and processing speed compared to occasional users.
O’Donnell et al. (2021)	America	Cross-sectional study	33 current cannabis users and 35 cannabis-non-users (18-35 years)	Users vs. non-users	Performance tasks and self-report	IGT, DD Task, PRLT, and BIS-11	Frequency and years of use examined; co-use/confounders not clearly reported	Cannabis users showed a greater preference for immediate vs. delayed rewards and self-reported impulsivity. No between-groups differences were observed in reversal learning. Frequency of past-month cannabis use and years of use did not predict decision making or impulsivity.
Willford et al. (2021)	America	Longitudinal cohort study	524 young adults (22 years)	Within-cohort exposure history	Standardized memory scale	Wechsler Memory Scale-III	Early initiation and developmental timing considered	Marijuana use at 15 age was associated with deficits in auditory-verbal, intelligence and memory in young adults. Current marijuana use was not associated with verbal memory function, when early initiation use was controlled.
Casey and Cservenka (2020)	America	Cross-sectional study	Thirty-three frequent marijuana users and 32 controls (18-22 years)	Frequent users vs. controls	Performance task and IQ test	Wechsler scale of intelligence-II and IGT	Sex differences examined; co-use/confounders not clearly reported	Marijuana users had greater risky decision-making than controls. Marijuana users had lower scores in vocabulary and matrix reasoning. The trend for sex differences in decision-making was not attributed to cannabis use.
Paige and Colder (2020)	America	Prospective longitudinal study	387 adolescents (11–12 years)	Within-cohort early-use levels over time	Self-report proxy	National Youth Survey, Achenbach System of Empirically Based Assessment, EATQ-R and ATQ	Questionnaire-based control outcomes; co-use/confounders not clearly reported	High level of early marijuana use at ages 12–14 significantly predicted low levels of attentional and inhibitory control at ages 18–21.
Ross et al. (2020)	America	Quasi-experimental co-twin control study	856 adolescents and young adults (16-28 years)	Co-twin comparison	Standardized tests and computerized EF tasks	CIDI-SAM, WAIS-III, The Raven Progressive Matrices and, 9 computerized EF tasks	Genetic/shared-environment confounding addressed directly	Minimal evidence for causal effects of cannabis on intelligence and executive functions (response inhibition, working memory and mental shifting). Correlations observed between cannabis use and cognition are explained by genetic factors.
Duperrouzel et al. (2019)	America	Longitudinal study	401 adolescents (14-17 years)	Within-cohort exposure gradients	Standardized battery and decision-making tasks	PHQ, WRAT-4, DASS-21, toxicology testing, DUHQ, IGT, GDT, CT, Decision-Making Latent Variable, WMS-IV, CVLT-II, Episodic Memory Latent Variables	Mood, academic achievement, toxicology, and drug history assessed	↑ baseline levels of cannabis use were associated with both poorer decision-making and episodic memory performance at baseline, cross-sectionally. ↑ cannabis use was associated with declines in immediate memory performance, but not delayed memory. Decision-making performance did not predict change in cannabis use frequency.
Kloft et al. (2019)	Europe	Field study	53 cannabis users under acute influence of cannabis, 53 sober but regular cannabis users, and 53 controls (18-30 years)	Intoxicated users vs. sober regular users vs. controls	Experimental memory paradigm	DRM	Acute vs. sober regular exposure differentiated; co-use/confounders not clearly reported	Intoxicated and sober cannabis consumers falsely recognized more unrelated items than control participants. Higher memory accuracy in cannabis-naive controls.
Lahanas and Cservenka (2019)	America	Cross-sectional study	28 frequent marijuana users and 33 healthy controls (18–22 years)	Frequent users vs. healthy controls	Performance task and IQ test	Modified-WCST, and WASI-II	Alcohol and nicotine examined; no significant influence beyond marijuana use reported	Marijuana users had lower scores in vocabulary and matrix reasoning. Impaired cognitive flexibility in heavy marijuana users. No significant influence of substance use (alcohol or nicotine) other than marijuana use on cognitive flexibility. Poorer cognitive flexibility in frequent marijuana users may relate to recent (past 30-day) marijuana use.
Laspada et al. (2019)	America	Analytical cross-sectional study	654 young adults (21 years)	Users vs. non-users by recency	Computerized memory tasks	ISL and ISLR	Exposure recency differentiated; co-use/confounders not clearly reported	Lower memory and learning scores in past-30 days marijuana users. Past-year cannabis users showed lower accuracy in memory tests, but not in verbal learning tests.
Pillersdorf and Scoboria (2019)	America	Cross-sectional study	47 cannabis users and 52 non-user controls (17-31 years)	Users vs. non-user controls	Autobiographical memory task and questionnaires	SCEPT, CUDIT-R, AUDIT, PHQ-9, BAI and FAB Fading affect bias protocol	Alcohol, anxiety, depression, and cannabis-use severity measured	Cannabis use was associated with higher rates of fading affect for unpleasant events. Cannabis user showed a reduced memory specificity.

AUDIT, Alcohol Use Disorders Identification Test; PHQ-9, Patient Health Questionnarie-9; ESPAD, Questionnaire on the use of sychoactive substances; FTND, The Fagerstrom Test for Nicotine Dependence; SDQ, Strengths and Difficulties Questionnaire; WISC, Wechsler Intelligence Scale for Children; WAIS, Wechsler Adult Intelligence Scale; SDT, Stromberg Dexterity Test; THC, delta-9-tetrahidrocannabinol; CBD, cannabidiol; NIH, National Institutes of Health; ABCD Study, Adolescent Brain Cognitive Development Study; GDT, Games of Dice Task; IGT, Iowa Gambling Task; BSRT, Buschke’s Selective Reminding Test; WCST, Wisconsin Card Sorting Test; MoCA, Montreal cognitive assessment; WM, Working Memory; PCC, Posterior Cingulate Cortex; CUDIT, Cannabis Use Disorder Test; BDI, Beck Depression Inventory; DSM, Diagnostic and Statistical Manual of Mental Disorders; DNA, Deoxyribonucleic Acid; RAVLT, Rey Auditory Verbal Learning Test; CI, Cociente Intellectual; BVN, Neuropsychological assessment battery for the developmental age; MT Trials-Advanced 3, Assessment test of reading skills, reading comprehension, and mathematical skills; CIDI, Composite International Diagnostic Interview; SAM, Substance Abuse Module; EF, executive functions; THC, tetrahidrocannabinol; WRAT-4, The Wide Range Achievement Test 4; DASS-21, Depression Anxiety and Stress Scale-21; DUHQ, Drug Use History Questionnaire; CT, Cups Tasks; CVLT-2, California Verbal Learning Test, Second Edition; MJ, Marihuana; HC, healthy controls; SCEPT, Sentence Completion for Events From the Past; PHQ-9, Patient Health Questionnaire; BAI, Beck Anxiety Inventory; EMO, Emotional Word-Emotional; PSI, Psychotomimetic States Inventory; SCID-II, The Drug Use History Questionnaire and Structured Clinical Interview for DSM-IV; CVLT-II, California Verbal Learning Test II; DD, delay discounting; PRLT, the Probabilistic Reversal Learning Task; BIS-11, Barratt Impulsiveness Scale; TAP, Test of Attentional Performance; STAI, State–Trait Anxiety Inventory; ISLT, The Cogstate International Shopping List Test; ISLR, The Cogstate International Shopping List Test-Recall; ATQ, Adult Temperament Questionnaire; EATQ-R, Early Adolescent Temperament Questionnaire; FAB, Fading affect bias protocol; and DRM, The Deese–Roediger–McDermott paradigm.

### Evaluation of methodological quality and certainty of evidence

Three researchers (LV-H, FC and VJV-B) independently appraised study quality, and disagreements were resolved by consensus. These procedures were used to strengthen rigor and transparency, rather than to guarantee reproducibility.

The methodological quality of individual studies was assessed using the Mixed Methods Appraisal Tool (MMAT), version 2018 (Hong et al., 2018). For non-randomized quantitative studies, MMAT evaluates whether: (1) participants are representative of the target population; (2) measurements are appropriate; (3) outcome data are complete; (4) confounders are adequately considered; and (5) the intervention or exposure occurred as intended. In accordance with MMAT guidance, we report the criteria met by each study and use these ratings to characterize methodological strengths and limitations.

Certainty in the body of evidence was considered separately using the Grading of Recommendations Assessment, Development and Evaluation (GRADE) approach. GRADE was used at the level of the overall evidence for each cognitive domain, taking into account risk of bias, inconsistency, indirectness, imprecision, and publication bias. A GRADE Evidence Assessment was carried out following the guidelines proposed by Aguayo-Albasini et al. (2014).

Based on the classification criteria established by Villanueva-Blasco et al. (2024a, 2024b), studies were categorized according to methodological quality assessed with the MMAT. The classification was as follows: (a) Very low or null evidence, no effectiveness reported, or quasi-experimental or randomized controlled trials (RCTs) with MMAT scores ≤40%; (b) Low, some positive effects observed in quasi-experimental studies with MMAT scores ≤40%; (c) Moderate low, positive effects from quasi-experimental studies with MMAT scores of 60%; (d) Moderate high, evidence of effectiveness from quasi-experimental studies with MMAT ≥80%, or RCTs with scores between 60 and 80%; and (e) High, clear effectiveness shown in one or more quasi-experimental evaluations or RCTs with MMAT ≥80%.

## Results

From the initial 2,251 records, 724 duplicates were manually removed, leaving 1,527 studies for title and abstract screening. After title and abstract screening to eliminate studies irrelevant to the aim, 44 articles remained. Finally, a thorough review of the full text of the articles was carried out, applying the inclusion and exclusion criteria. In total, 22 studies were included. Table 1 presents the characteristics of the included studies.

Five reports could not be retrieved in full text despite repeated attempts through institutional access, publisher webpages, and supplementary search routes. These records were therefore excluded prior to full-text eligibility assessment.

### Quality assessment

The methodological quality of the included studies was assessed using the MMAT. The majority of studies were quantitative non-randomized studies and were therefore evaluated using the criteria applicable to that domain. Lawn et al. (2022a) is presented separately because it was the only randomized controlled trial identified in the review; accordingly, it was assessed using the MMAT criteria specific to randomized studies rather than those for non-randomized designs. The complete quality appraisal is reported in Appendix Tables A2, A3.

Most of the included studies were quantitative non-randomized designs, together with one randomized controlled trial. Overall, methodological quality was heterogeneous. The most frequent limitations involved incomplete control of confounding variables, variation in exposure definition, and differences in the sensitivity and comparability of cognitive outcome measures.

Importantly, methodological quality at the level of individual studies should not be interpreted as equivalent to certainty of evidence. Small exploratory studies may satisfy several MMAT criteria while still contributing limited certainty due to imprecision. For this reason, the revised manuscript distinguishes more clearly between study-level methodological appraisal and synthesis-level certainty of evidence.

### Study characteristics

**Figure 2:**
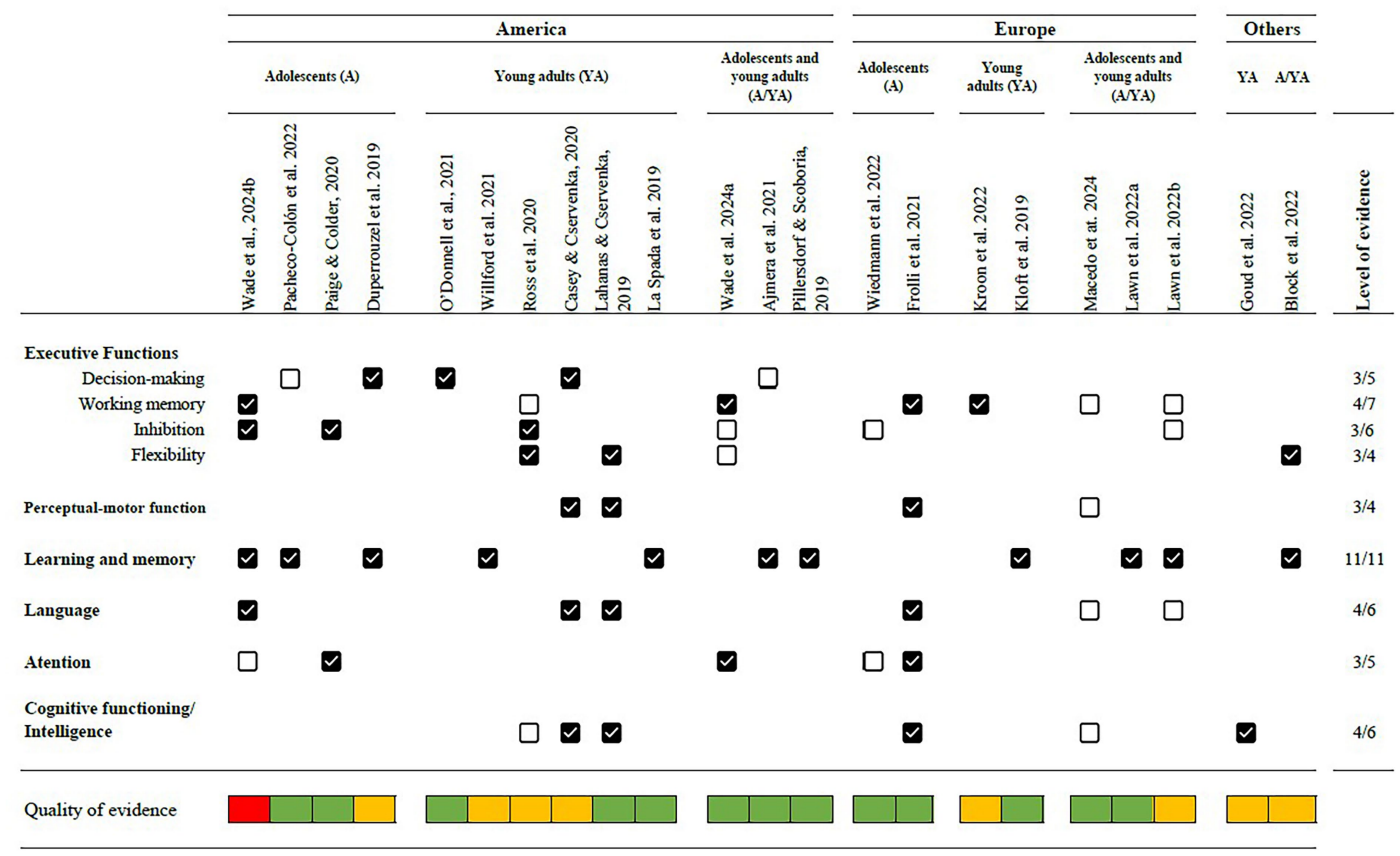
Review of evidence for effect of cannabis use on cognitive function as a function of geographical area and age. Level of evidence was expressed as the numbers of studies evidencing a negative effect of cannabis use against the total number of studies. The color coding system employed to denote the quality of the evidence is as follows: red, yellow and green represent low, moderate-high and high methodological quality of the study, respectively (see text for more details).

Table 1 and Figure 2 summarize the main characteristics of the 22 studies included in this review; 7 studies were conducted in Europe, 13 in the Americas, 1 in Asia, and 1 included participants from both Europe and the Americas. No eligible studies from Oceania were identified in the final sample. Studies from the Americas were predominantly conducted in the United States, with one study from Chile and one cross-continental study including both American and Dutch adolescents.

With respect to age-group classification, studies were synthesized as involving adolescents, young adults, or mixed adolescent/young-adult samples, depending on the developmental framing and age range of the recruited sample. Because age boundaries varied across studies, this categorization was used to structure the synthesis rather than to impose a universal age cutoff across all studies.

The cognitive domains examined were diverse. Decision-making was evaluated in five studies (Ajmera et al., 2021; Casey and Cservenka, 2020; Duperrouzel et al., 2019; O’Donnell et al., 2021; Pacheco-Colón et al., 2022), while cognitive flexibility was assessed in four (Block et al., 2022; Lahanas and Cservenka, 2019; Ross et al., 2020; Wade et al., 2024a). Six studies explored inhibitory control (Lawn et al., 2022a; Paige and Colder, 2020; Ross et al., 2020; Wade et al., 2024a, 2024b; Wiedmann et al., 2022). Memory and/or learning outcomes were assessed in 11 studies (Ajmera et al., 2021; Block et al., 2022; Duperrouzel et al., 2019; Kloft et al., 2019; Laspada et al., 2019; Lawn et al., 2022a; Lawn et al., 2022b; Pacheco-Colón et al., 2022; Pillersdorf and Scoboria, 2019; Wade et al., 2024b; Willford et al., 2021). Attention was evaluated in four studies (Frolli et al., 2021; Wade et al., 2024a, 2024b; Wiedmann et al., 2022), visuoperceptual abilities in five (Casey and Cservenka, 2020; Frolli et al., 2021; Lahanas and Cservenka, 2019; Wiedmann et al., 2022), and six articles examined language-related functions (Casey and Cservenka, 2020; Frolli et al., 2021; Lahanas and Cservenka, 2019; Lawn et al., 2022a; Macedo et al., 2024; Wade et al., 2024b).

A major source of heterogeneity was the non-equivalence of the tasks used to operationalize the same nominal cognitive domain. Episodic memory was assessed with measures such as the CVLT-II, WMS-based tasks, Picture Sequence Memory, Buschke’s Selective Reminding Test, shopping-list paradigms, and false-memory protocols. Working memory was measured with n-back paradigms, List Sorting, and Wechsler-derived indices. Inhibitory control was assessed using the Flanker task, Stroop-type tasks, and, in some studies, broader self-regulatory or attentional/inhibitory control indicators rather than laboratory-based inhibition tasks. Decision-making was examined through paradigms such as the Iowa Gambling Task, Game of Dice Task, and Cups Task. Because these measures differ in modality, psychometric sensitivity, and the cognitive subprocesses they capture, direct cross-study comparisons should be interpreted cautiously.

Not all cognitive measures were commensurate in depth or specificity. Some studies used brief global screening tools, such as the Montreal Cognitive Assessment (MoCA), whereas others used multidomain neuropsychological batteries or fine-grained performance-based tasks. Accordingly, findings derived from brief screening measures were interpreted as broad indicators of global cognitive functioning and were not weighted in the same way as domain-specific findings from more comprehensive cognitive assessments.

### Impact of cannabis use on neurocognitive functioning considering the geographical area of study

In Europe, several studies consistently report that cannabis use negatively affects verbal learning and episodic memory (Lawn et al., 2022a; Lawn et al., 2022b; Wiedmann et al., 2022). In one study using a false-recognition paradigm, intoxicated and regular cannabis users showed greater false recognition than controls. We therefore interpret this finding as increased susceptibility to memory distortion or altered memory monitoring, rather than as evidence of a generalized cognitive deficit (Kloft et al., 2019). Working memory deficits have been found among both chronic and occasional users (Frolli et al., 2021; Kroon et al., 2024), with more pronounced impairments as task demands increase (Kroon et al., 2024). However, not all findings are aligned. A longitudinal study found no significant working memory impairment in occasional users before or after cannabis initiation (Macedo et al., 2024), and no spatial working memory differences were noted among chronic users (Lawn et al., 2022a).

Regarding inhibitory control, two studies conducted in adolescents found no significant impairments linked to cannabis use (Lawn et al., 2022a; Wiedmann et al., 2022), nor any association between level of use and performance (Wiedmann et al., 2022). Attention-related findings are mixed: some studies reported no relationship (Wiedmann et al., 2022), while others identified slower processing speed in chronic users as compared to non-consumers (Frolli et al., 2021). Additional European data show impairments in visuo-perceptual reasoning and verbal comprehension in chronic users (Frolli et al., 2021), though not in occasional ones (Macedo et al., 2024).

In the Americas, both longitudinal studies (Ajmera et al., 2021; Duperrouzel et al., 2019; Pacheco-Colón et al., 2022; Willford et al., 2021) and cross-sectional research (Laspada et al., 2019; Pillersdorf and Scoboria, 2019; Wade et al., 2024b) consistently associate cannabis use with impairments in verbal learning and episodic memory. Executive function deficits are also widely documented. Frequent use is linked to reduced cognitive flexibility (Lahanas and Cservenka, 2019; Ross et al., 2020), poorer inhibitory control (Paige and Colder, 2020; Ross et al., 2020; Wade et al., 2024b), and increased risk-taking during decision-making (Casey and Cservenka, 2020; Duperrouzel et al., 2019; O’Donnell et al., 2021). These associations persist even after adjusting for variables like sex, age of onset, and race, although some studies report only modest effects (Wade et al., 2024a). Working memory deficits also correlate with recent cannabis use (Wade et al., 2024a, 2024b). However, genetic factors may partly explain these findings (Ross et al., 2020). Deficits in language and reasoning are also observed in frequent users compared to healthy controls (Casey and Cservenka, 2020; Lahanas and Cservenka, 2019), with heavier use linked to poorer receptive language and attention (Wade et al., 2024b).

Across both regions, long-term memory impairments are reported, with effects more prominent in American users (Block et al., 2022). In Asia, the only study reported cognitive deficits in chronic users relative to non-users, with partial recovery after three months of abstinence, though generalization is limited due to the focus on heavy users (Goud et al., 2022).

### Impact of cannabis use on neurocognitive functioning as a function of the age (adolescents vs. young adults)

The cognitive effects of cannabis use differ significantly depending on the user’s age and the cognitive domain assessed.

Episodic and working memory are among the most consistently affected functions, particularly in adolescents. Several studies report reduced performance in verbal and visual episodic memory tasks among adolescent cannabis users (Duperrouzel et al., 2019; Lawn et al., 2022b; Pacheco-Colón et al., 2022; Wade et al., 2024b). Working memory deficits have also been observed, especially in chronic or frequent adolescent users (Frolli et al., 2021). In young adults, memory impairments are also evident. Kroon et al. (2024) found decreased working memory performance in frequent users, and Laspada et al. (2019) observed lower scores in recent users as compared to non-users. Notably, Willford et al. (2021) emphasized that memory deficits in young adults were more strongly associated with early cannabis use than with current consumption. A placebo-controlled trial found no age-related differences in the acute effects of cannabis on verbal memory (Lawn et al., 2022b). Moreover, both acute and regular users appear more prone to false memories than control participants, indicating possible impairments in memory accuracy (Kloft et al., 2019).

Executive function deficits, particularly in inhibitory control and decision-making, are more pronounced in adolescents. Early cannabis use has been linked to reduced attentional and inhibitory control in early adulthood (Paige and Colder, 2020), while other studies have reported lower inhibitory control (Wade et al., 2024b) and impaired decision-making in adolescent users (Duperrouzel et al., 2019). However, Pacheco-Colón et al. (2022) found that decision-making performance did not significantly predict cannabis initiation or escalation.

Among young adults, frequent cannabis use has been associated with greater impulsivity, preference for immediate rewards, and riskier decision-making (Casey and Cservenka, 2020; O’Donnell et al., 2021). Nevertheless, a twin study by Ross et al. (2020) found limited evidence for a causal relationship, suggesting that genetic factors may contribute to the observed effects.

Visuospatial reasoning and general intelligence also seem more affected in adolescents cannabis users. Frolli et al. (2021) reported significant declines in IQ, processing speed, and visuospatial reasoning among chronic adolescent users. In contrast, Macedo et al. (2024) found no significant differences between regular and non-user young adults. However, lower IQ scores have been observed in cannabis users aged 18–22 in other studies (Casey and Cservenka, 2020; Lahanas and Cservenka, 2019).

### Quality of the evidence on the effects of cannabis use on neurocognitive functioning

The quality of evidence on the neurocognitive effects of cannabis varies depending on the cognitive domain evaluated, the age group of the participants (adolescents or young adults), and the geographical region of the study (Tables 2, 3).

**Table 2:** Quality of the evidence from the studies included in this systematic review on neurocognitive effects of cannabis.

Geographic area	Sample population	Study	Design of study	MMAT	Quality of evidence
America	Adolescents	Wade et al. (2024b)	Cross-sectional study	40	Low
		Pacheco-Colón et al. (2022)	Longitudinal study	100	High
		Paige and Colder (2020)	Longitudinal study	100	High
		Duperrouzel et al. (2019)	Longitudinal study	80	Moderate-high
	Young adults	O’Donnell et al. (2021)	Cross-sectional study	100	High
		Willford et al. (2021)	Longitudinal cohort study	80	Moderate-high
		Ross et al. (2020)	Quasi-experimental Co-twin control study	60	Moderate-high
		Casey and Cservenka (2020)	Cross-sectional study	80	Moderate-high
		Lahanas and Cservenka (2019)	Cross-sectional study	100	High
		Laspada et al. (2019)	Cross-sectional study	100	High
	Adolescents and young adults	Wade et al. (2024a)	Cross-sectional study	100	High
		Ajmera et al. (2021)	Prospective longitudinal study	100	High
		Pillersdorf and Scoboria (2019)	Cross-sectional study	100	High
Europe	Adolescents	Wiedmann et al. (2022)	Cross-sectional study	100	High
		Frolli et al. (2021)	Cross-sectional study	100	High
	Young adults	Kroon et al. (2024)	Cross-sectional study	80	Moderate-high
		Kloft et al. (2019)	Field study	100	High
	Adolescents and young adults	Macedo et al. (2024)	Longitudinal study	100	High
		Lawn et al. (2022a)	Randomized controlled trial	100	High
		Lawn et al. (2022b)	Cross-sectional study	80	Moderate-high
Asia	Young adults	Goud et al. (2022)	Observational case–control study	80	Moderate-high
America and Europe	Adolescents and young adults	Block et al. (2022)	Cross sectional study	80	Moderate-high

**Table 3:** Summary of the quality level of evidence by age and continent.

Cognitive function		Adolescents			Young adults		
		Europe	America	Asia	Europe	America	Asia
Memory	Episodic memory	High	High	Moderate	High	Moderate-high	Moderate
	Working memory	High	Low	-	Moderate-high	Moderate	-
Executive functions	Decision-making	-	Moderate-high	-	-	Moderate-high	-
	Impulsivity	-	Moderate	-	-	Moderate-high	-
	Inhibitory control	Moderate	High	-	Low	Moderate	-
Attention		High	Low	-	-	-	-
Language		Moderate	Moderate	-	Low	Moderate-high	-
Visuospatial reasoning		High	-	-	Moderate-high	Moderate	-
Intelligence		High	Moderate-high	-	Moderate-high	Moderate	-

Robust evidence from longitudinal studies in the Americas (Duperrouzel et al., 2019; Pacheco-Colón et al., 2022) and cross-sectional studies in Europe (Wiedmann et al., 2022) consistently links adolescent cannabis use to impairments in verbal learning and episodic memory when compared with non-using peers. Additional studies from Europe (Frolli et al., 2021) and the Americas (Wade et al., 2024b) have reported working memory deficits in adolescents users relative to controls, though the latter presents methodological limitations. Among young adults, moderate-to-high quality research shows memory impairments compared with controls in American and European samples (Kloft et al., 2019; Laspada et al., 2019; Willford et al., 2021). In Asia, one study reported that episodic memory deficits in chronic users may improve after abstinence (Goud et al., 2022). In Europe, working memory impairments were also observed in young adult cannabis users (Kroon et al., 2024), although a U.S. twin study suggested minimal causal effects (Ross et al., 2020).

Regarding inhibitory control, longitudinal findings from America indicate that early cannabis use predicts poorer inhibitory functioning in adulthood (Paige and Colder, 2020), supported by cross-sectional data (Wade et al., 2024b). In contrast, no deficits were observed in European adolescents (Wiedmann et al., 2022), though chronic adolescent use was linked to impaired planning (Frolli et al., 2021). While American adolescents cannabis users showed decision-making impairments (Duperrouzel et al., 2019), these were not predictive of use escalation (Duperrouzel et al., 2019; Pacheco-Colón et al., 2022). In American young adults, frequent users exhibited impulsivity and impaired decision-making relative to non-using controls (Casey and Cservenka, 2020; O’Donnell et al., 2021), along with deficits in cognitive flexibility (Lahanas and Cservenka, 2019; Ross et al., 2020), though not assessed in European samples.

Findings on attention are inconclusive. While some studies reported processing speed and attention deficits in adolescent chronic users (Frolli et al., 2021), others found no such impairments (Wade et al., 2024b; Wiedmann et al., 2022). No studies focused exclusively on attention in young adult users.

Language-related research is scarce. In America, young adult users showed poorer vocabulary performance (Casey and Cservenka, 2020; Lahanas and Cservenka, 2019), and adolescents had slightly weaker receptive language scores (Wade et al., 2024b) compared with controls. In Europe, adolescent cannabis use was linked to reduced verbal comprehension (Frolli et al., 2021), though other studies reported no significant differences (Lawn et al., 2022a; Macedo et al., 2024).

Lastly, impairments in visuospatial reasoning and intelligence have been reported across both continents (Casey and Cservenka, 2020; Frolli et al., 2021; Lahanas and Cservenka, 2019), although an American twin study suggests these may reflect genetic rather than cannabis-related effects (Ross et al., 2020).

## Discussion

Across the studies published between 2019 and 2024, the most reproducible neurocognitive signal associated with cannabis use was poorer memory performance, particularly in episodic and working memory. Evidence for executive functions was more heterogeneous and depended strongly on developmental stage, exposure profile, study design, and task selection. More consistent associations tended to emerge in adolescent samples, in longitudinal studies, and in studies using more sensitive or repeated measures of exposure. Accordingly, the present findings support a cautious interpretation in which cannabis-related neurocognitive differences are most evident under conditions of earlier onset, heavier exposure, and greater measurement sensitivity, rather than as uniform deficits across all users and all domains.

### Evidence according to geographical area

Scientific evidence on the neurocognitive effects of cannabis differs by region, influenced by variability in findings, methodologies, usage patterns, and study quality.

In the Americas, 13 studies, many with longitudinal designs and moderate to high methodological quality, report consistent cognitive impairments linked to cannabis use (Duperrouzel et al., 2019; Pacheco-Colón et al., 2022; Paige and Colder, 2020; Willford et al., 2021). These include deficits in episodic and verbal memory, working memory, inhibitory control, decision-making, impulsivity, and language-related abilities (Casey and Cservenka, 2020; Lahanas and Cservenka, 2019; Laspada et al., 2019; Wade et al., 2024b; Willford et al., 2021). Heavy use has been particularly associated with poor cognitive performance (Casey and Cservenka, 2020; Lahanas and Cservenka, 2019; Wade et al., 2024a). However, some findings suggest that these effects may be driven by shared genetic vulnerabilities rather than direct causality (Ross et al., 2020).

In Europe, findings are more mixed. Stronger evidence supports memory impairments, especially in chronic adolescent users (Frolli et al., 2021; Lawn et al., 2022b; Wiedmann et al., 2022). Other studies report increased false memories (Kloft et al., 2019) and decreased visuoperceptual reasoning (Frolli et al., 2021). However, some found no significant differences in inhibitory control or spatial working memory (Lawn et al., 2022a; Macedo et al., 2024), likely reflecting variations in usage frequency and type. The high prevalence of cannabis-tobacco co-use in Europe (Villanueva-Blasco et al., 2024a) may also influence results and complicate attribution of effects solely to cannabis.

In Asia, only one study was identified. It showed general cognitive impairments in chronic users, with partial recovery after abstinence (Goud et al., 2022). While methodologically rigorous, its generalizability is limited due to sample size and exclusion of heavy users.

Overall, the geographical synthesis suggests that cannabis-related neurocognitive disorders are reported more consistently in the Americas and more heterogeneously in Europe, and these differences are likely to reflect variations related to cannabis consumption patterns, as well as contextual and methodological factors.

Although cannabis use is generally associated with cognitive impairments, regional differences in findings have emerged. Some European studies reported no deficits in inhibitory control or attention (Lawn et al., 2022a; Wiedmann et al., 2022), contrasting with American research showing impairments in these domains (Paige and Colder, 2020; Wade et al., 2024a, 2024b). These discrepancies may reflect cultural influences, differing consumption patterns (exclusive use vs. cannabis-tobacco co-use vs. cannabis-alcohol concurrent use), or variations in assessment tools. For example, while Macedo et al. (2024) found no cognitive differences between light users and non-users in Europe, American studies identified impairments even in moderate users (Ajmera et al., 2021; Macedo et al., 2024; Wade et al., 2024a, 2024b).

Regional differences should not be attributed to cannabis–tobacco co-use alone. Cannabis products differ across markets in potency and composition, with substantial increases in THC and reductions in CBD reported in recent years, and these changes may shape cumulative exposure and neurocognitive outcomes (European Union Drugs Agency, 2024). In addition, regional variation in product type, route of administration, and patterns of repeated use may further influence cognitive findings, particularly when studies do not report dose, product characteristics, or biochemical verification consistently (European Union Drugs Agency, 2024; Villanueva-Blasco et al., 2024a). The regional evidence base also differed methodologically: studies from the Americas more often used longitudinal designs and frequency-sensitive exposure measures (Ajmera et al., 2021; Duperrouzel et al., 2019; Pacheco-Colón et al., 2022; Willford et al., 2021), whereas European studies were more often cross-sectional and relied on more heterogeneous task selection and mixed exposure profiles (Kloft et al., 2019; Kroon et al., 2024; Lawn et al., 2022a; Macedo et al., 2024; Wiedmann et al., 2022). Region should therefore be interpreted as a contextual lens that may partly capture broader differences in exposure, design, and measurement, rather than as a simple geographical effect per se.

Although both continents report episodic memory deficits, Block et al. (2022) observed more severe impairments in American participants. This suggests that cognitive outcomes may be shaped not only by individual or task-related factors but also by broader sociocultural variables. Differences in cannabis consumption styles, particularly the prevalence of exclusive cannabis use or the frequent concurrent use of alcohol and cannabis in the Americas (Substance Abuse and Mental Health Services Administration, 2025) versus co-use with tobacco in Europe (European Union Drugs Agency, 2024; Villanueva-Blasco et al., 2024a), may partly explain the greater cognitive impairments observed in American samples.

Future research should further consider these region-specific consumption patterns, as they could contribute to explaining the cross-continental differences in cognitive outcomes. To better disentangle region-specific effects, future studies should incorporate objective biomarkers of exposure when possible. Tobacco exposure can be assessed using cotinine, including plasma or serum cotinine, whereas cannabis exposure can be verified through THC and/or cannabinoid metabolites measured in blood, urine, or oral fluid (Antunes et al., 2023). Because these markers were not consistently collected in the included studies, particularly in European samples, geographic distinctions based on presumed consumption patterns should be interpreted cautiously.

Overall, these findings underscore the importance of considering regional patterns, cannabis formulations, and cultural context in interpreting cannabis’s neurocognitive effects.

### Differences in evidence by age

The more pronounced pattern observed in adolescents is likely multifactorial rather than attributable to age alone. Adolescence is a period of ongoing maturation of prefrontal, hippocampal, and fronto-limbic systems, as well as of endocannabinoid-related neurodevelopmental processes, which makes this stage biologically plausible as a window of increased vulnerability to cannabis-related cognitive effects (Cabeen et al., 2020; Lu and Mackie, 2021; Mena et al., 2013; Ross and Levy, 2023). At the same time, the apparent age gradient may also reflect differences in the available evidence base, since adolescent studies in this review more often captured earlier onset, developmental trajectories, or school-age samples (Duperrouzel et al., 2019; Pacheco-Colón et al., 2022; Paige and Colder, 2020; Wade et al., 2024b), whereas young-adult studies more frequently involved cross-sectional cohorts with broader heterogeneity in use patterns and more variable findings (Casey and Cservenka, 2020; Kroon et al., 2024; Lahanas and Cservenka, 2019; O’Donnell et al., 2021). This interpretation is also consistent with prior reviews indicating that age of onset is a key moderator of cannabis-related neurocognitive risk, although the evidence remains insufficient to infer a simple age-only effect (Duperrouzel et al., 2020; Fonseca-Pedrero et al., 2020; Gorey et al., 2019). Moreover, because some studies have reported minimal or reversible structural effects of cannabis exposure, the present findings should be interpreted cautiously and not as definitive proof that age alone explains the observed differences (Scott et al., 2019).

Among adolescents, consistent deficits have been reported in episodic memory (Pacheco-Colón et al., 2022; Wade et al., 2024b), inhibitory control (Paige and Colder, 2020; Wade et al., 2024b), and processing speed (Frolli et al., 2021), particularly among early-onset or chronic users. Longitudinal data indicate that early cannabis use predicts poorer inhibitory control and attentional performance in early adulthood (Paige and Colder, 2020), suggesting long-term neurodevelopmental consequences. Chronic adolescent users have also shown reductions in IQ (Frolli et al., 2021), while occasional users exhibit milder impairments.

In young adults, although cognitive deficits are also observed, findings are more variable. Working memory impairments (Goud et al., 2022; Kroon et al., 2024) and poor decision-making (Casey and Cservenka, 2020; O’Donnell et al., 2021) have been reported, though studies involving light users often find no significant differences (Lawn et al., 2022a; Macedo et al., 2024). Notably, several impairments are more closely tied to early cannabis initiation during adolescence than to current use (Willford et al., 2021). Additionally, young adults often show elevated impulsivity (O’Donnell et al., 2021) and riskier decision-making (Casey and Cservenka, 2020), likely due to ongoing maturation of emotional regulation and behavioral control mechanisms during this developmental stage. Changes in cannabis potency and cannabinoid composition over time may also contribute to variability in observed neurocognitive outcomes and should be considered in future longitudinal research (European Union Drugs Agency, 2024).

The present findings are broadly consistent with previous systematic reviews, although they also refine them. Prior syntheses have suggested that cannabis use is associated with small-to-moderate disadvantages across several cognitive domains, while also noting uncertainty regarding whether adolescent-onset users are uniquely more impaired than adult users (Duperrouzel et al., 2020; Gorey et al., 2019). Our review partially converges with these conclusions: memory-related alterations remain the most consistent finding, adolescent samples show a somewhat clearer concentration of working-memory and inhibitory-control results, and executive-function findings remain mixed. At the same time, the present review extends previous work by focusing on more recent studies and by incorporating a structured regional synthesis, which suggests that apparent age effects may be intertwined with contextual differences across study settings rather than reflecting a simple developmental gradient alone.

Overall, the age-stratified synthesis indicates that cannabis-related neurocognitive differences are detected more consistently in adolescents than in young adults, although this pattern likely reflects both developmental vulnerability and differences in the available evidence base.

### Quality of evidence and limitations

Several factors beyond age group and geographical setting likely contributed to the differential and sometimes contradictory findings observed across studies. These include differences in study design, with longitudinal studies better capturing developmental trajectories and temporal associations than cross-sectional designs (Ajmera et al., 2021; Duperrouzel et al., 2019; Pacheco-Colón et al., 2022; Willford et al., 2021 vs. Frolli et al., 2021; Kroon et al., 2024; O’Donnell et al., 2021; Wade et al., 2024a, 2024b; Wiedmann et al., 2022), as well as frequent limitations in sample size and representativeness (Ajmera et al., 2021; Pillersdorf and Scoboria, 2019; Wiedmann et al., 2022). Heterogeneity in cannabis-user profiles also complicated interpretation, since the included studies variably examined occasional, light, frequent, chronic, recent, intoxicated, or abstinent users, often with inconsistent abstinence windows and limited characterization of dose, potency, or route of administration (Ajmera et al., 2021; Frolli et al., 2021; Goud et al., 2022; Kloft et al., 2019; Lawn et al., 2022a; Macedo et al., 2024; Wade et al., 2024a, 2024b). In addition, the control of alcohol and nicotine/tobacco co-use was uneven across studies (Lahanas and Cservenka, 2019; Pillersdorf and Scoboria, 2019), and potentially relevant participant characteristics, such as psychopathology, sex-related vulnerability, academic functioning, and family context, were not consistently addressed (Ross et al., 2020; Wade et al., 2024a, 2024b). Marked heterogeneity was also evident in the operationalization of cognitive domains: ostensibly similar constructs were assessed using non-equivalent measures ranging from standardized batteries to brief tasks and self-report indicators (Frolli et al., 2021; Kloft et al., 2019; Lahanas and Cservenka, 2019; Paige and Colder, 2020; Wade et al., 2024a, 2024b). This lack of methodological and measurement uniformity may partly explain why some studies detected impairments whereas others did not.

The methodological quality of the included studies was found to be moderate to high. Longitudinal designs (Ajmera et al., 2021; Duperrouzel et al., 2019; Macedo et al., 2024; Pacheco-Colón et al., 2022; Paige and Colder, 2020; Willford et al., 2021) proved valuable for identifying temporal associations and, in some cases, supporting causal interpretations. Several studies also controlled for key sociodemographic, genetic, and environmental factors (Duperrouzel et al., 2019; Macedo et al., 2024; Pacheco-Colón et al., 2022; Ross et al., 2020; Wade et al., 2024a), strengthening the validity of their conclusions on cannabis-related cognitive impairment.

Nonetheless, various methodological limitations were identified. Many studies employed cross-sectional designs (Frolli et al., 2021; Kroon et al., 2024; O’Donnell et al., 2021; Wade et al., 2024a, 2024b; Wiedmann et al., 2022), limiting causal inference and complicating the differentiation between cannabis effects and pre-existing vulnerabilities. Small or non-representative samples were also common (Ajmera et al., 2021; Pillersdorf and Scoboria, 2019; Wiedmann et al., 2022), reducing generalizability.

There was significant variability in the cognitive assessment methods used. Some studies employed standardized neuropsychological batteries (Frolli et al., 2021; Wade et al., 2024b), while others relied on brief tasks or self-reports of questionable validity (Kloft et al., 2019; Lahanas and Cservenka, 2019), hindering comparability across studies.

An additional source of discrepancy is the lack of homogeneity in cannabinoid dose and composition across studies and overtime. Cannabis use classifications were inconsistent (Ajmera et al., 2021; Frolli et al., 2021), and most studies did not measure THC dosage, potency, or route of administration (Goud et al., 2022; Wade et al., 2024a), a crucial factor in a market that increases potency practically every year. Cannabis potency and composition have changed substantially, with increases in THC and reductions in CBD (European Union Drugs Agency, 2024). This introduces uncertainty in the interpretation of cognitive effects, particularly among individuals exposed during adolescence. This lack of harmonization (e.g., absent reporting of THC concentration, absolute dose, product type, or route) likely inflates between-study heterogeneity and may obscure dose–response relationships, thereby complicating cross-study comparisons and meta-analytic synthesis. Future work should adopt common potency/dose metrics and minimum reporting standards, along with biochemical verification.

Crucially, many studies failed to consider factors such as mental health, academic achievement, family dynamics, and sex differences, despite emerging evidence of sex-specific vulnerability. Additionally, few addressed cannabis-tobacco interactions or distinguished between exclusive and combined use, a relevant gap in regions like Europe where co-use is prevalent.

A final limitation concerns the interpretation of methodological quality and certainty of evidence. Although many studies met several MMAT criteria, this should not be interpreted as meaning that the corresponding evidence is necessarily precise or conclusive. Small samples, residual confounding, cross-sectional designs, and heterogeneity in cognitive measures all reduce confidence in strong causal interpretations.

Overall, cannabis use appears to be linked to poorer neurocognitive performance, especially in memory. However, because the studies were highly heterogeneous and potential confounding factors were not always fully controlled, these findings should not be interpreted as strong evidence of causality.

### Practical implications

This systematic review highlights key implications for policy and research. Consistent evidence of impairments in verbal memory, inhibitory control, and decision-making, especially in adolescents, underscores the urgency of early prevention strategies to delay cannabis initiation and reduce use. Evidence-based interventions in schools and communities should address cannabis’s neurocognitive impact during brain development. Programs must be culturally adapted to regional consumption patterns, given differences between the Americas and Europe. Mental health and education professionals should be trained to detect early cognitive decline. In clinical settings, rehabilitation plans must include cognitive assessments, particularly for young users with early, chronic, or poly-substance use histories. A special focus on cannabis-tobacco interactions should be considered. Future research should focus on high-quality, multicenter longitudinal studies with standardized cognitive measures and attention to usage patterns. Expanding research into underrepresented regions, including Asia, Africa, and Latin America, is essential to generate globally relevant evidence.

## Conclusion

This systematic review confirms that cannabis use, especially when initiated in adolescence or sustained frequently, is linked to neurocognitive impairments, particularly in episodic and working memory, inhibitory control, and decision-making, with more consistent and severe effects in adolescents. Regional differences likely stem from variations in use patterns, study quality, and contextual factors. Notably, distinguishing exclusive from combined cannabis-tobacco use is essential, especially in Europe. These findings support early prevention, targeted interventions, and multicenter longitudinal research to enhance global understanding.

## Data Availability

The raw data supporting the conclusions of this article will be made available by the authors, without undue reservation.
