# Supplementary material for: Neuropsychological effects of cannabis use by continent and age: a systematic review
Source: Front Psychol. 2026 May 28;17:1728743. doi: 10.3389/fpsyg.2026.1728743 (PMC13253619; doi:10.3389/fpsyg.2026.1728743)
Supplement: Supplementary file 1 [file Table_1.DOCX]

**Table A1.** Search strategy implemented to conduct the systematic review.

| Nº Step | **Search strategy** |
| --- | --- |
| **WOS** | |
| S1 | AB=(Cannabis OR Marihuana OR Marijuana OR THC) |
| S2 | AB= (Cogniti* OR Neuropsycholog* OR Attention OR “Working memory” OR “Executive Function” OR Shifting OR Switching OR “Decision making” OR visuospatial) |
| S3 | AB=(adolescent OR “Young adult”) |
| S4 | S1 AND S2 AND S3 |
| S5 | Publication date Last 5 years |
| **PUBMED** | |
| S1 | (Cannabis[Title/Abstract] OR marihuana[Title/Abstract] OR marijuana[Title/Abstract] OR THC[Title/Abstract]) OR (cannabis[MeSH Terms]) |
| S2 | (cognit*[Title/Abstract] OR neuropsycholog*[Title/Abstract] OR attention[Title/Abstract] OR "working memory"[Title/Abstract] OR shifting[Title/Abstract] OR switching[Title/Abstract] OR "decision making"[Title/Abstract]) OR (cognition OR "executive function" OR memory OR attention OR neuropsychology[MeSH Terms]) |
| S3 | ((adolescent[Title/Abstract] OR "young adult"[Title/Abstract]) OR (adolescent OR "young adult"[MeSH Terms])) |
| S4 | S1 AND S2 AND S3 |
| **SCOPUS** | |
| S1 | (TITLE-ABS-KEY ( cannabis OR marihuana OR marijuana OR thc)) |
| S2 | (TITLE-ABS-KEY (cogniti* OR neuropsycholog* OR attention OR "working memory" OR "executive function*" OR shifting OR switching OR "decision making")) |
| S3 | (TITLE-ABS-KEY ( adolescent OR "young adult")) |
| S4 | S1 AND S2 AND S3 |

| **OPEN GREY** | |
| --- | --- |
| S1 | Cognition AND cannabis AND (adolescent OR "young adult") |

**Table A2.** Quality appraisal of quantitative non-randomized trials

|  | **Methodological quality criteria** | | | | | | |
| --- | --- | --- | --- | --- | --- | --- | --- |
| **Studies** | **S1** | **S2** | **1** | **2** | **3** | **4** | **5** |
| Macedo et al. (2024) | Yes | Yes | Yes | Yes | Yes | Yes | Yes |
| Wade et al. (2024a) | Yes | Yes | Yes | Yes | Yes | Yes | Yes |
| Wade et al. (2024b) | Yes | Yes | Yes | Yes | No | No | No |
| Block et al. (2022) | Yes | Yes | Yes | Yes | Yes | No | Yes |
| Goud et al. (2022) | Yes | Yes | Yes | Yes | Yes | No | Yes |
| Kroon et al. (2024) | Yes | Yes | Yes | Yes | Yes | No | Yes |
| Lawn et al. (2022b) | Yes | Yes | Yes | Yes | Yes | No | Yes |
| Pachecho-Colón et al. (2022) | Yes | Yes | Yes | Yes | Yes | Yes | Yes |
| Wiedmann et al.(2022) | Yes | Yes | Yes | Yes | Yes | Yes | Yes |
| Ajmera et al. (2021 | Yes | Yes | Yes | Yes | Yes | Yes | Yes |
| Frolli et al. (2021) | Yes | Yes | Yes | Yes | Yes | Yes | Yes |
| O’Donnell et al. (2021) | Yes | Yes | Yes | Yes | Yes | Yes | Yes |
| Willford et al. (2021) | Yes | Yes | Yes | Yes | No | Yes | Yes |
| Ross et al. (2020) | Yes | Yes | No | Yes | Yes | No | Yes |
| Casey y Cservenka (2020) | Yes | Yes | No | Yes | Yes | Yes | Yes |
| Paige y Colder (2020) | Yes | Yes | Yes | Yes | Yes | Yes | Yes |
| Duperrouzel et al. (2019) | Yes | Yes | No | Yes | Yes | Yes | Yes |
| Kloft et al. (2019) | Yes | Yes | Yes | Yes | Yes | Yes | Yes |
| Lahanas & Cservenka (2019) | Yes | Yes | Yes | Yes | Yes | Yes | Yes |
| Laspada et al. (2019) | Yes | Yes | Yes | Yes | Yes | Yes | Yes |
| Pillersdorf & Scoboria (2019) | Yes | Yes | Yes | Yes | Yes | Yes | Yes |
| Questions: (S1) Are there clear research questions?; (S2) Do the collected data allow to address the research questions?; (1) Are the participants representative of the target population?; (2) Are measurements appropriate regarding both the outcome and intervention (or exposure)?; (3) Are completed outcome data?; (4) Are the cofounders accounted for in the design and analysis; (5) During the study period, is the intervention administered (or exposure occurred) as intended? | | | | | | | |

**Table A3.** Quality appraisal of randomized controlled trials

|  | **Methodological quality criteria** | | | | | | |
| --- | --- | --- | --- | --- | --- | --- | --- |
| **Studies** | **S1** | **S2** | **1** | **2** | **3** | **4** | **5** |
| Lawn et al. (2022a) | Yes | Yes | Yes | Yes | Yes | Yes | Yes |
| Questions: (S1) Are there clear research questions?; (S2) Do the collected data allow to address the research questions?; (1) Is randomization appropriately performed?; (2) Are the groups comparable at baseline?; (3) Are there complete outcome data?; (4) Are outcome assessors blinded to the intervention provided?; (5) Did the participants adhere to the assigned intervention? | | | | | | | |
